# First-Line Combination of R-CHOP with the PDE4 Inhibitor Roflumilast for High-Risk DLBCL

**DOI:** 10.3390/cancers16223857

**Published:** 2024-11-18

**Authors:** Adolfo E. Diaz Duque, Pedro S. S. M. Ferrari, Purushoth Ethiraj, Carine Jaafar, Zhijun Qiu, Kenneth Holder, Mathew J. Butler, Gabriela Huelgas-Morales, Anand Karnad, Patricia L. M. Dahia, Ricardo C. T. Aguiar

**Affiliations:** 1 Division of Hematology and Medical Oncology, Mays Cancer Center, University of Texas Health Science Center San Antonio, San Antonio, TX 78229, USA; 2 Department of Pathology, University of Texas Health Science Center San Antonio, San Antonio, TX 78229, USA; 3 South Texas Veterans Health Care System, Audie Murphy VA Hospital, San Antonio, TX 78229, USA

**Keywords:** lymphoma, clinical trial, phosphodiesterase, PI3K, angiogenesis

## Abstract

Background: Diffuse large B-cell lymphoma (DLBCL) is a common and often fatal malignancy. The standard-of-care immunochemotherapy, R-CHOP, cures only about 60% of DLBCL patients. Improving this cure rate will likely require the effective translation of basic biology knowledge into clinical activities. We previously identified the cyclic-AMP/phosphodiesterase 4 (PDE4) axis as an important modulator of lymphomagenic processes. We also showed that the FDA-approved PDE4 inhibitor roflumilast can suppress B-cell receptor (BCR) signals, phosphoinositide 3-kinase (PI3K) activity and angiogenesis. These data suggested that combining roflumilast with R-CHOP may be beneficial in DLBCL. Methods: We conducted a single-center, single-arm, open-label, phase 1 study of roflumilast in combination with the standard of care, R-CHOP (Ro+R-CHOP), in pathologically proven, treatment-naïve, high-risk DLBCL patients. Results: Ro+R-CHOP was safe, and at a median follow-up time of 44 months, 70% of patients were alive and disease free (median OS not reached, PFS 44% (95% CI, 21–92). In this pilot series, we found that the addition of roflumilast suppressed PI3K activity in peripheral blood mononuclear cells, and VEGF-A secretion in the urine. We also encountered preliminary evidence to suggest that the Ro+R-CHOP combination may be particularly beneficial to patients diagnosed with high-risk genetic subtypes of DLBCL, namely MCD and A53. Conclusions: These initial findings suggest that roflumilast may be an alternative agent able to inhibit BCR/PI3K activity and angiogenesis in DLBCL, and that the testing of Ro+R-CHOP in a larger series of genetically characterized tumors is warranted. This study was registered at ClinicalTrials.gov, number NCT03458546.

## Simple Summary

Diffuse large B-cell lymphoma (DLBCL) is a common and often fatal cancer type. Despite recent progress in our understanding of DLBCL biology, the translation of this knowledge into clinical initiatives has lagged, and the first-line treatment for this tumor type, the immunochemotherapy R-CHOP, has been the same for more than two decades. We previously identified the cyclic-AMP/phosphodiesterase 4 (PDE4) axis as a critical modulator of B-cell receptor (BCR) signals and PI3K activity in DLBCL. Pre-clinically and clinically, we confirmed that PDE4 inhibition suppressed PI3K activity, and downstream to it, angiogenesis in the lymphoma microenvironment. Here, we report on a phase 1 clinical trial that combines the PDE4 inhibitor roflumilast with R-CHOP in treatment-naïve DLBCL patients. We show that this combination is safe, active, and inhibits PI3K activity and VEGF-A levels. Further, we preliminarily identified the genetic subtypes of DLBCL that may be especially vulnerable to this new drug combination.

## 1. Introduction

In diffuse large B-cell lymphoma (DLBCL), the response rate to first-line therapy has remained largely unchanged since the combination of anti-CD20 antibody, rituximab (R), and multi-agent chemotherapy (CHOP—cyclophosphamide, doxorubicin hydrochloride, oncovin and prednisone) became the standard of care more than 20 years ago [1,2]. Regrettably, only approximately 60% of DLBCL patients are cured with R-CHOP, and improving this cure rate is an unmet medical need. Recently, the incorporation of the antibody drug conjugate Polatuzumab Vedotin into front-line therapy was shown to modestly improve the progression-free survival (PFS) of DLBCL patients, but it had no impact on overall survival (OS) [3]. The field has also made progress on at least two other fronts. First, CD19 CAR-T cell therapy for relapsed/refractory (R/R) DLBCL has improved disease-free and overall survival in this subset of patients [4]. Second, the additional dissection of the genetics of DLBCL has expanded our understanding of the molecular basis of its clinical heterogeneity and highlighted the need to rationally individualize treatment towards an improved cure rate [5,6].

We previously found that the gene encoding the cyclic-AMP (cAMP)-hydrolyzing enzyme phosphodiesterase 4B (*PDE4B*) is expressed at significantly higher levels in biopsies from fatal than cured DLBCL patients [7,8]. This finding made immediate sense because cAMP signaling is inhibitory in B lymphocytes. Thus, an aberrantly high expression of PDE4B would blunt this natural constraint on B-cell activation and proliferation [9]. Following this discovery, we used preclinical in vitro and in vivo models to map the breadth of the cAMP/PDE4 axis effects on DLBCL biology. We found that PDE4B-expressing DLBCLs display higher SYK-BTK-PIK3 activity downstream of the B-cell receptor (BCR), and that the FDA-approved PDE4 inhibitor (PDE4i) roflumilast (https://pubchem.ncbi.nlm.nih.gov/compound/Roflumilast, accessed on 6 November 2024) suppresses these signals and promotes lymphoma cell death [7,10,11,12,13]. These compelling preclinical data led us to test the PDE4i roflumilast in combination with high-dose corticosteroid for patients with relapsed/refractory mature B-cell malignancies. In this recently completed phase 1 trial, roflumilast was found to be safe and active [14]. These data agreed with our preclinical findings indicating that PDE4i can improve or restore glucocorticoid sensitivity [12]. In addition, in this first clinical study, we demonstrated that roflumilast’s effect correlated with the suppression of PI3K activity [12]. This finding was particularly important because although aberrant PI3K signals are an integral part of DLBCL pathogenesis, the clinical testing of PI3K inhibitors has been characterized by low activity and high toxicity [15].

We have also uncovered the unexpected role of cAMP/PDE4 in modulating lymphoma angiogenesis. In this instance, by controlling the expression of cell intrinsic pro-angiogenic factors (VEGF-A, MYC and HIF1α) or by directly inhibiting endothelial cells in the tumor microenvironment, we found that PDE4 inhibition meaningfully suppressed lymphoma angiogenesis [16,17]. This observation is relevant because angiogenesis is a known marker of a poor outcome in DLBCL, but attempts to combine R-CHOP with the bevacizumab were marred by cardiovascular toxicity [18,19,20,21,22,23,24]. Thus, the testing of novel pharmacological strategies with potential anti-angiogenic properties is warranted in DLBCL.

Here, we describe the testing of the combination of roflumilast (Ro) with R-CHOP (Ro+R-CHOP) in a pilot cohort of treatment-naïve patients with DLBCL of non-GCB (germinal center B-cell like) origin. In this phase 1 trial, we found that roflumilast is safe and active in high-risk DLBCLs. Notably, we detected the significant suppression of VEGF-A levels and PI3K activity in patients receiving Ro+R-CHOP. In addition, we that the addition of roflumilast to R-CHOP may improve the response rate of genetic subtypes of DLBCL characterized by a poor outcome [5,6,25].

## 2. Methods

Study design. We conducted a single-center, phase 1, open-label, single-arm study of the PDE4 inhibitor roflumilast in combination with R-CHOP in pathologically proven NOS (not otherwise specified) DLBCL patients with a non-GCB subtype [26] and who had not received prior systemic therapy for lymphoma.

Disease burden was assessed at baseline with FDG-PET/CT scans (fludeoxyglucose-18 positron emission tomography/computed tomography). The baseline left ventricular ejection fraction (LVEF) was measured to assess pre-treatment cardiac function. Additional testing was performed to establish the disease characteristics and stage, including the sampling of bone marrow and cerebrospinal fluid (if clinically indicated). In addition to standard laboratory monitoring, and considering the putative anti-angiogenic properties of roflumilast, the serum levels of troponin and B-type natriuretic peptide (BNP) were monitored, as biomarkers for cardiac toxicity. Urine samples were collected for the measurement of VEGF-A levels prior to cycle 1 (baseline), cycle 3 (C3) and cycle 6 (C6). Peripheral blood mononuclear cells (PBMC) were obtained at baseline and prior to C3, as described [27]. Adverse events (AE) were assessed and documented at each visit. A patient health questionnaire (PHQ-9) was administered prior to each cycle to screen for the new onset of depression, which could be related to treatment. The patients’ response was evaluated after all planned treatments were completed, or sooner if the patient discontinued treatment before the sixth cycle. An FDG-PET/CT was obtained six to eight weeks after the completion of chemotherapy to assess the best response to treatment, according to the “Revised Response Criteria for Malignant Lymphoma” [28]. By design, the use of interim FDG-PET or CT scans prior to the completion of therapy was discouraged, unless there was clinical deterioration necessitating early evaluation. LVEF was also re-assessed after treatment was completed. This study was registered at ClinicalTrials.gov, number NCT03458546.

Objectives and Endpoints. The primary objective was the assessment of safety and tolerability. The secondary objective was the anti-tumor activity of Ro+R-CHOP. The exploratory objectives included the impact of roflumilast added to R-CHOP on VEGF-A levels and PI3K activity, and an investigation of the association between the genetic subtypes of DLBCL and clinical outcomes following Ro+R-CHOP. The primary endpoint was the assessment of AE and serious adverse events (SAEs), graded according to the NCI—Common Terminology Criteria for Adverse Events (CTCAE) version 4.03. The secondary endpoints were the estimation of complete response (CR), as defined by the normalization of FDG-PET uptake (Deauville score of 1 to 3) for all target lesions.

Patient eligibility. This study enrolled adult patients aged 18 years or older who had non-GCB NOS-DLBCL and had not received prior systemic therapy for lymphoma. The inclusion criteria were as follows: Eastern Cooperative Oncology Group (ECOG) performance status of 0–2, life expectancy of ≥3 months, Ann Arbor stage II-IV, measurable disease with lesions with a long axis of ≥1.5 cm by CT imaging, and at least one FDG-avid lesion according to FDG-PET scan. Additional inclusion criteria were as follows: LVEF of at least 45% according to either echocardiography or radionucleotide angiography, creatinine clearance ≥ 30 mL/min according to the Cockcroft–Gault formula, total bilirubin ≤ 1.5 × upper limit of normal (ULN) (unless indirect bilirubin was elevated due to Gilbert’s syndrome or hemolysis), AST and ALT ≤ 3 × ULN, platelet count ≥ 50,000/µL, ANC ≥ 1000/µL, and hemoglobin ≥ 8 g/dL.

This study did not include patients with an allergy or intolerance to roflumilast, with any active malignancy other than DLBCL, with a diagnosis of high-grade B-cell lymphoma with rearrangements of MYC, BCL2 and/or BCL6, with a prior allogeneic bone marrow transplant within 12 months of the screening date, with a prior autologous stem cell transplant within 6 months of the screening date, with active central nervous system (CNS) involvement by lymphoma, or patients with a HIV-positive status, hepatitis B or C infection, a history of depression or other psychiatric illnesses. In addition to the study cohort, patients with a diagnosis of NOS-DLBCL treated with the standard of care (SOC), R-CHOP, and who consented to be part of the anonymized biorepository at our institution were enrolled as a “control” group for the analysis of the biomarker in urine and blood (Supplemental Table S1). Ro+R-CHOP and SOC R-CHOP patients came from the same South Texas catchment area, were seen in the same institution/clinic, and were treated by the same care team. This study was conducted according to the Declaration of Helsinki, was approved by the University of Texas Health Science at San Antonio’s institutional review board, and all participants provided written informed consent.

Treatment. All patients received R-CHOP therapy at standard doses (rituximab 375 mg/m^2^, cyclophosphamide 750 mg/m^2^, doxorubicin 50 mg/m^2^, and vincristine 1.4 mg/m^2^ capped at 2 mg, all given intravenously on day 1, and 100 mg of prednisone orally on days 1 through 5), repeated every 21 days for 6 cycles. All patients were scheduled to receive a fixed oral dose of a 500 microgram (µg) roflumilast tablet per day for the 21 days of each cycle. The first dose was given on the day of the first R-CHOP treatment. The continuation of roflumilast on subsequent cycles was contingent on troponin and BNP levels, as well as an assessment of unexpected toxicity, and/or evidence of disease refractoriness.

Patients with known involvement of the bone marrow, peripheral blood, orbit, nasal or paranasal sinus, or testis, or with more than one extra nodal site of disease with elevated LDH, received prophylactic central nervous system chemotherapy with 4–8 doses of 12 mg methotrexate, administered intrathecally. Patients with bulky disease, defined as at least one tumor mass ≥ 10 cm of the largest dimension, as well as serum LDH above the upper limit of normal, received tumor lysis syndrome prophylaxis per institutional guidelines.

Measurements of urinary VEGF-A levels. Urine samples were obtained from all patients enrolled in the Ro+R-CHOP trial, as well as from the control group of DLBCL patients receiving SOC R-CHOP (Supplemental Table S1) (total, n = 17). Urinary, instead of circulating (plasma/serum), VEGF-A was measured due to the experience of the SWOG group, in the context of lymphoma trials. In those instances, multiple variables associated with phlebotomy hampered the accurate measurement of plasma values of VEGF, contrary to its robust quantification in the urine [24]. This observation was confirmed by others, with the suggestion that in cancer patients, the plasma/serum levels of VEGF may be primarily related to platelet-derived and not tumor-derived secretion [29,30]. The urinary VEGF-A levels were measured using the AuthentiKine™ human VEGF ELISA kit (Proteintech Group Inc., Rosemont, IL, USA), according to the manufacturer’s instructions. The VEGF-A levels in the urine were normalized by corresponding urine creatinine levels using a creatinine (urinary) colorimetric assay kit (Cayman Chemical, Ann Arbor, MI, USA), as described [31].

PI3-kinase activity assay. Whole peripheral blood was collected from the two cohorts of patients referenced above. PBMCs were isolated by Ficoll–Hypaque processing, and the samples were stored in liquid nitrogen for future use. Cell lysates were obtained for the characterization of PI3K activity, which was measured using a PI3-kinase Activity ELISA kit (Echelon Biosciences Inc., Salt Lake City, UT, USA), as we reported [14].

Genetic analysis. DNA was isolated from buccal mucosa and DLBCLs biopsies (formalin fixed paraffin-embedded–FFPE) from seven patients enrolled in the Ro+R-CHOP study (for three patients, no FFPE blocks were available for DNA isolation), as described [32]. Whole-exome sequencing (WES) was performed in matched germline and tumor samples from seven patients using the Agilent v6.0 library kit, and these were paired-end 150 bp sequenced on an Illumina NovaSeq (Illumina Inc., San Diego, CA, USA). The average depth of the tumor samples was 410 (177–563, range), and that for germline specimens was 317 (228–438, range). Reads were mapped to the human genome (hg38) using BWA, and somatic variants were called from the aligned .bam files using the GATK4 workflow performed using a Snakemake pipeline, as described [33,34]. This process included base quality score recalibration, local realignment, somatic short mutation calling with Mutect2, and somatic copy number variations (CNV) using the GATK4 somatic-cnv workflow. A panel of normal genomes (PON) was constructed using GATK4, and this included 33 exome samples from the 100 Genomes Project, along with the seven matched germline samples of the current cohort, resulting in a total of 40 samples. The PON was used as a reference for somatic mutations and copy number variant calls in the tumor samples following the GATK4 best practices parameters. Finally, the available data on BCL2 and BCL6 translocation, along with the somatic mutation calls and CNV information, were inputted into the LymphGen 1.0 tool [25] for the classification of the lymphoma samples according to recently defined genetic subtypes [5].

Safety, efficacy statistical analyses. Safety was evaluated by the incidence, severity, and type of adverse events that occurred during the treatment and follow-up periods using NCI’s CTCAE, as described above. Although the primary aim of this study was to establish safety and tolerability, the rate of objective response to treatment was recorded and assessed by the investigators using standard clinical and imaging criteria. After treatment was completed, PFS and OS were assessed and recorded, as determined by the investigators during routine clinical follow-up visits. Given its exploratory nature, and the small cohort of 10 patients, the statistical analysis was only descriptive. The statistical significance of the differences in VEGF-A levels and PI3K activity were tested using a two-sided Student’s *t*-test; when appropriate, equal variance was calculated with an F-test. These analyses were performed in the Graph-Pad Prism9 software package (Graph-Pad Prism 10.2.3). *p* values < 0.05 were considered statistically significant.

## 3. Results

Patients’ demographic and baseline characteristics. Sixteen patients were screened, and ten patients met the eligibility criteria for enrollment. Sixty percent were male, 50% were Hispanic, the median age at diagnosis was 60 years old (33–79), 100% had ECOG 0–1, all tumors had a non-GCB immunophenotype, and 50% were double MYC/BCL2 expressors (Table 1). Fifty percent of the patients enrolled were Lugano stage 2, and the other 50% were stage 3–4. The Revised International Prognostic Index (R-IPI) score was ≥2 in 70% of the patients. CNS prophylaxis was implemented in 50% of the patients. The trial was conducted between June 2018 and November 2020. Patients still enrolled in the study (n = 7) were followed for 36 to 60 months (median 44 months); the data were locked up on 1 November 2023.

Safety. Standard doses of R-CHOP were delivered safely with the addition of 500 µg of roflumilast on a daily basis. The adverse events were not dissimilar from those identified in historical SOC R-CHOP series. In brief, one patient (10%) experienced neutropenia grade 3 and two patients (20%) had neutropenia grade 2; anemia grade 1–2 occurred in two patients (20%). Non-hematologic AE included anorexia grade 1 (40%), diarrhea grade 1 (50%), headache grade 2–3 (60%), nausea grade 1–2 (30%) and grade 3 (10%), and weight loss grade 1–2 (40%). Eight patients received roflumilast for at least three R-CHOP cycles. Two patients received roflumilast for one or two cycles only. In all instances in which roflumilast was stopped, it was stopped because of an asymptomatic grade 1 increase in BNP and/or troponin, as per protocol. Although asymptomatic, patients with elevated BNP and/or troponin were monitored more closely. These patients were subjected to electrocardiograms and transthoracic echocardiograms, which showed a preserved left ventricular ejection fraction, and no abnormal or pathologic findings. The asymptomatic elevation of BNP and/or troponin was attributed to the use of anthracyclines, as recently reported [35]. Notably, the cardiotoxicity detected in the clinical trials that tested the antiangiogenic agent bevacizumab in combination with R-CHOP was severe, being primarily heart failure [23], and was markedly distinct from the asymptomatic elevation of BNP and/or troponin found in the Ro+R-CHOP-treated patients. We concluded that adding roflumilast to R-CHOP does not increase the potential risk of cardiotoxicity associated with anthracycline.

Efficacy. Of the ten patients who received Ro+R-CHOP, nine were evaluable for efficacy (one patient who received a single roflumilast cycle was excluded from the efficacy analysis, as per protocol). Six patients (66%) remained alive and in complete remission (disease free) at the time that the analysis was locked (median follow-up of 44 months, range 33–60) (Table 2). The median OS has not been reached. In brief, at 12 and 24 months, the OS was 89% (95% CI, 70–100%) and 67% (95% CI, 42–100%), respectively, and the PFS was 56% (95% CI, 31–99%) and 44% (95% CI, 21–92), respectively. Three patients died of their disease, after achieving a partial response (PR) and failing or declining second-line treatment; the median survival for this R/R group was 15 months (range 12–24). Two of the six evaluable patients currently alive and free of disease had early relapses 5 and 9 months after complete response (CR), but were successfully rescued with second-line therapy. These patients remain free of disease 33 and 54 months after second remission (Table 2). In a previously reported cohort of non-GCB DLBCLs treated with R-CHOP, the 24-month PFS and OS were 28% (95% CI, 15–51%) and 46% (95% CI, 30–69%), respectively [36]. Thus, we cautiously suggest that the outcome with Ro+R-CHOP is, at a minimum, not inferior to historical data for SOC R-CHOP [1,3,36].

**Figure 1:**
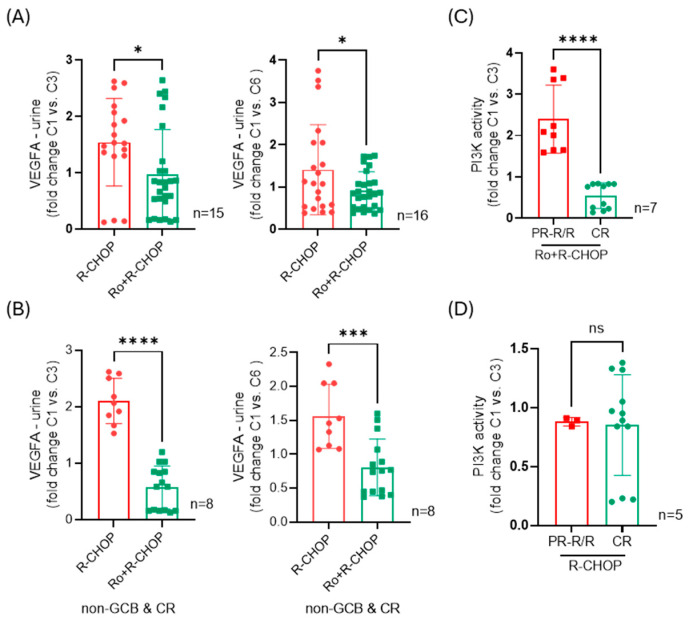
VEGF-A quantification and PI3K activity in DLBCL patients treated with SOC R-CHOP or Ro+R-CHOP. (**A**) Left to right, fold change in urinary VEGF-A levels from baseline (before cycle 1—C1) to after the completion of two or five treatment cycles (at the start of cycle 3 or cycle 6, C3 or C6); nine Ro+R-CHOP- and six or seven R-CHOP-treated patients were included in these analyses. (**B**) Left to right, fold change in urinary VEGF-A levels from baseline (before cycle 1—C1) to after the completion of two or five treatment cycles (at the start of cycle 3 or cycle, C3 or C6); only non-GCB DLBCL patients and complete responders (CRs) were included in these analyses, with five Ro+R-CHOP- and three R-CHOP-treated patients. (**C**) Fold change in PI3K activity measured in the PBMCs of patients treated with Ro+R-CHOP, comparing the baseline (before cycle 1—C1) to after the completion of two treatment cycles (at the start of cycle 3—C3). Three patients with PR-R/R (partial responder–relapsed/refractory) who died of their disease are compared to four patients who achieved CR and remain free of disease (materials were not available for UPN#1, a complete responder and long-term survivor). (**D**) Fold change in PI3K activity measured in the PBMCs of patients treated with SOC R-CHOP, comparing the baseline (before cycle 1—C1) to after the completion of two treatment cycles (at the start of cycle 3—C3). Two patients in this series did not reach CR, and materials were not available for one of them. Data are mean ± SD. All data points quantified are shown (three replicates for each patient); *p* values are from two-sided Student’s *t*-test, * < 0.05, *** < 0.001, **** < 0.0001.

Impact of roflumilast on VEGF-A secretion. Pre-clinically, in vivo and in vitro, we have shown that the genetic or pharmacologic inhibition of PDE4 suppresses angiogenesis by acting on both tumor cells and the endothelium [2,9,16,17]. These effects are in part associated with the suppression of VEGF-A secretion by the tumor cells [17]. To validate this observation in human DLBCL patients, we quantified the VEGF-A levels in the urine of patients receiving Ro+R-CHOP and compared it to the values found in a contemporary local cohort of DLBCL patients treated with SOC R-CHOP. Adding roflumilast to the immunochemotherapy regimen significantly suppressed urinary VEGF-A, quantified early (after two cycles) or late (after five cycles) in the course of treatment (Figure 1A). Notably, the difference between Ro+R-CHOP and R-CHOP was even more marked when the comparison was stricter and included only data from non-GCB DLBCL patients who were complete responders (Figure 1B). We concluded that the addition of roflumilast to the first-line treatment of non-GCB DLBCL patients decreases VEGF-A in the urine, a surrogate for the inhibition of angiogenesis.

Suppression of PI3-K activity aligns with response to Ro+R-CHOP. Pre-clinically, in vivo and in vitro, we have shown that the genetic or pharmacologic inhibition of PDE4 suppresses the activity of multiple kinases downstream of the BCR receptor, including PI3K [7,10,11,12,17]. In our first clinical trial of R/R mature B-cell malignancies, we detected an improved response in cases with the suppression of PI3K activity [14]. Here, we validated this observation and found that after two cycles of Ro+R-CHOP, a significantly lower PI3K activity was detected in the PBMCs of patients who achieved CR in comparison to patients with PR or refractory disease (Figure 1C). By comparison, in patients receiving SOC R-CHOP, PI3K activity was not different between responders and non-responders (Figure 1D). We concluded that in this small series of DLBCL patients treated with Ro+R-CHOP, the suppression of PI3K activity early in the treatment plan may predict the long-term response.

Genetic subtypes and clinical response. Paired exome sequencing was completed, and the LymphGen algorithm [25] was used to define the genetic subtype of DLBCL in seven of the 10 patients treated with the Ro+R-CHOP combination. This classification system has been proposed to uncover unique pharmacological vulnerabilities and to have potential value in precision medicine trials. In our series, using the core and extended LymphGen tool [25], six tumors were classified, and one tumor remained unassigned (other). Two cases were defined as A53, two as BN2, one as composite (MCD + A53) and one as EZB (Table 2, Supplemental Tables S2 and S3, Supplemental Figure S1), a distribution close to that previously defined for non-GCB (ABC) DLBCLs [5,6,25]. Notably, the three patients categorized as having A53 and/or MCD DLBCLs, subtypes known to be associated with a poor outcome, were complete responders and are free of disease at 44, 55 and 60 months. The data from this small series suggest that the addition of roflumilast to R-CHOP may benefit high-risk patients.

## 4. Discussion

There is broad agreement in the field that to improve the cure rate in cancer, we need to better understand the biology of disease and identify the specific vulnerabilities amendable to therapeutic intervention. The testing of the PDE4 inhibitor roflumilast in combination with R-CHOP for the treatment of DLBCL patients follows this precept. In earlier work, we identified that *PDE4B* is one of the differentially expressed genes in fatal vs. cured DLBCL [7,8]. We followed that discovery by meticulously mapping the multiple mechanisms by which increasing cAMP levels, a direct consequence of PDE4 inhibition, suppress lymphoma cell growth and modulate the tumor microenvironment [11,12,13,16,17]. We conducted an earlier clinical trial which confirmed that the PDE4i roflumilast is safe in R/R mature B-cell malignancies [14]. Herein, we report on the use of this agent in treatment-naïve DLBCL patients. These clinical initiatives, built on the repurposing of roflumilast [37,38]) for the treatment of B-cell cancers, led to the preliminary validation of PI3K and VEGF-A as biomarkers of response. In the current report, we also tentatively linked the activity of roflumilast to the subtypes MCD and A53, which have a poor genetic outcome. We hypothesize that these tumors may be particularly vulnerable to PDE4 inhibition because they are driven by aberrant BCR activation (MCD and A53) and, potentially, increased angiogenesis (A53), two oncogenic processes countered by roflumilast.

The association between the DLBCL subtype A53 and angiogenesis has not been formally demonstrated, but P53 loss has been linked to angiogenesis in multiple cancer types [39,40,41]. Importantly, angiogenesis is a well-defined marker of a poor outcome in DLBCL, possibly irrespective of genetic subtyping [18,19,20,21,22,23,24]. Anti-angiogenic strategies were tested in DLBCL in large phase 2 and phase 3 trials of bevacizumab + R-CHOP. However, these trials were halted due to excessive cardiotoxicity [23,24], likely reflecting the synergistic toxicity of VEGF-A inhibition with anthracyclines. We postulate that the milder anti-angiogenic effect of roflumilast, which we showed earlier to act both on the lymphoma cell and the endothelium [16,17], overcomes this limitation. Further, roflumilast has been in fact suggested to decrease the risk of adverse cardiovascular events in COPD patients [37,38]. In agreement with this evidence, we did not detect clinically relevant cardiotoxicity in the Ro+R-CHOP trial. Instead, due to an abundance of caution, we strictly monitored markers of cardiotoxicity (troponin and BNP) and may have prematurely stopped roflumilast in a few cases. This is an important consideration, because doxorubicin elevates the levels of troponin and BNP in R-CHOP-treated DLBCL patients [35]. Together, the data from this pilot series suggest that cardiotoxicity is not a safety concern in patients treated with a combination of roflumilast and R-CHOP.

The ability of roflumilast to downmodulate the constitutively active BCR signals, a hallmark of a significant fraction of DLBCL cases [5], has been extensively shown in pre-clinical models, in vitro and in vivo. In these studies, we found that PDE4 inhibition, and the consequent increase in intracellular cAMP levels, can directly and indirectly inhibit SYK, BTK and PI3K activity [7,9,10,11,12,13,17]. Notably, in two clinical series, we confirmed that patients who respond to roflumilast-containing regimens show the significant suppression of PI3K activity in PBMCs [14]. Intriguingly, patients who did not respond to the Ro+R-CHOP combination displayed elevated PI3K activity at cycle 3 in comparison to the pre-treatment measurements. We attributed this finding to an increased tumor burden, although we cannot exclude the possibility that other asymptomatic inflammatory or infectious conditions were present at the time of PBMC collection. Attempts to inhibit BCR activity in DLBCL (and related B-cell tumors) with SYK and PI3K inhibitors have been characterized by limited activity and excessive toxicity [15,42,43,44,45]. The testing of the BTK inhibitor ibrutinib in combination with R-CHOP in DLBCL initially yielded negative results [46]. However, additional analysis suggested that this combination may be effective in the MCD subtype [47]. We postulate that roflumilast may be a better option to target the chronic active BCR signaling of DLBCL, the driver of the MCD subtype [25]. First, roflumilast simultaneously targets multiple kinases downstream of the BCR, perhaps similarly to dasatinib, as recently suggested in a pre-clinical examination [48]. Second, mutant populations with acquired resistance are less likely to emerge. Third, the extensive clinical use of roflumilast (in COPD settings [37]) confirms its excellent safety profile, suggesting that the well-described toxicity associated with SYK, BTK and PI3K inhibitors may be avoided if these enzymes are inhibited indirectly via the roflumilast-induced elevation of cAMP levels. The validation of these postulates awaits a large clinical trial in genetically defined DLBCL that compares Ro+R-CHOP to R-CHOP. Of note, we do not suggest, or envision, that roflumilast could be effective as a single agent for the treatment of DLBCL, but rather we propose it as an adjuvant to immunochemotherapy schemes, including R-CHOP or polatuzumab vedotin + R-CHP.

Our study has limitations, including the small sample size of the investigational cohort, the lack of a formal control group and the open-label design. However, considering the well-established safety profile of roflumilast, we opted to close enrollment after 10 patients were accrued, as we felt that the information obtained was sufficient to expedite the opening of a larger pivotal phase 2 trial that compares Ro+R-CHOP to R-CHOP in genetically selected subsets of DLBCL. Another limitation of the study relates to the measurement of PI3K activity, which has been confined to PMBCs, an unavoidable restriction considering the impracticality of obtaining serial lymphoma tissue (e.g., an affected lymph node) during treatment. Nonetheless, the data from this report, together with our findings in an earlier clinical trial [14], suggest that the quantification of PI3K in this sample type may be informative.

## 5. Conclusions

In conclusion, we showed that the Ro+R-CHOP combination is safe in treatment-naïve DLBCL patients, that the addition of roflumilast inhibited VEGF-A secretion in the urine and PI3K activity in the PBMCs, and that this new therapeutic scheme may be particularly active in specific genetic subsets of DLBCL. Together, these data indicate that testing Ro+R-CHOP as a first-line treatment in a larger series of genetically characterized DLBCLs is warranted.

## Figures and Tables

**Table 1:** Demographics and baseline characteristics of DLBCL patients in the Ro+R-CHOP trial.

UPN	Gender/Age	Ethnicity	ECOG PS	R-IPI	Lugano Stage	Double MYC/BCL2 Expressors
1	M/53	non-Hispanic	1	2	IIIB	NA
2	M/69	Hispanic	1	4	IIIE	Yes
3	F/79	Hispanic	1	2	IIB	Yes
4	M/33	Unknown	0	0	IIE	No
7	F/64	Hispanic	0	3	IV	Yes
9	M/67	non-Hispanic	1	1	IIE	No
11	M/49	non-Hispanic	0	NA	IV	No
12	F/72	Hispanic	0	2	II	No
13	F/55	non-Hispanic	0	1	IIE	Yes
14	M/56	Hispanic	0	2	IV	Yes

**Table 2:** Outcome and biomarker features of DLBCL patients in the roflumilast + R-CHOP trial.

UPN	Best Response	^&^ Outcome/Follow Up Months	LymphGen Classifier	VEGF Suppression	PI3K Suppression
1	CR	Alive/60	MCD/A53	Yes	ND
2	PR	Deceased/15	Other	Yes	No
3	CR	Alive/55	A53	Yes	Yes
4	CR *	Alive/54	EZB	No	No
7	CR	Alive/44	A53	Yes	Yes
9	CR	Alive/40	ND	No	Yes
11	PR	Deceased/12	BN2	Yes	No
12	CR ^#^	Alive/36	BN2	Yes	Yes
13	R/R ^^^	Alive/33	ND	ND	ND
14	PR	Deceased/17	ND	No	No

^&^, all patients alive are disease free; * early CNS relapse, rescued with auto-transplant; ^#^ patient not evaluable for roflumilast efficacy—single cycle of investigational agent; ^^^ Refractory, off protocol on cycle 4, rescued with 2nd line chemotherapy; ND—not done.

## Data Availability

Data included in this work is available upon request.

## Supplementary Materials

The following supporting information can be downloaded at: https://www.mdpi.com/article/10.3390/cancers16223857/s1, Figure S1: LymphoGen algorithm classification; Table S1: Demographics, baseline features and outcome of DLBCL patients treated with SOC R-CHOP. Table S2: Patient characteristics and LymphGen input and output features—core predictions. Table S3: Patient characteristics and LymphGen input and output features—extended predictions.
