# Supplementary material for: First-Line Combination of R-CHOP with the PDE4 Inhibitor Roflumilast for High-Risk DLBCL
Source: Cancers (Basel). 2024 Nov 18;16(22):3857. doi: 10.3390/cancers16223857 (PMC11592688; doi:10.3390/cancers16223857)
Supplement: Supplementary file 1 [file cancers-16-03857-s001.zip › cancers-3300795-supplementary.pdf]

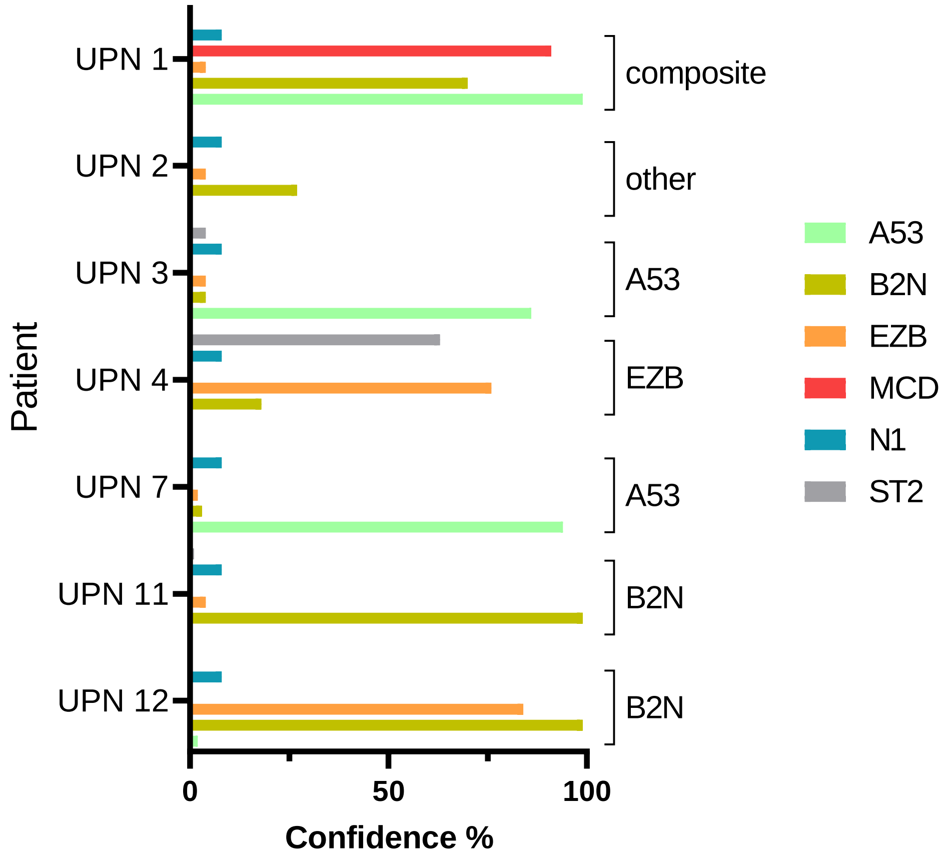

Supplemental Figure S1. LymphoGen algorithm classification. Paired next generation sequencing of seven patients treated with Ro+R-CHOP allowed their classification in defined genetic subtypes. Tumors that were core members (>90% probability) of more than one subtype are termed genetically composite, such as UPN#1, 91% MCD, 99% A53. In UPN#12, confidence in EZB "probability" in 84%, but 99% for B2N, thus not a composite DLBCL. Tumors in which subtype probabilities did not reach 50% were termed unassigned or other (UPN#2).

Supplemental Table S1. Demographics, baseline features and outcome of DLBCL patients treated with SOC R-CHOP

UPN	Gender/Age	COO/IHC	ECOG PS	R-IPI	Lugano Stage	Double MYC/BCL2 expressors	Best response
17	M/74	GCB	1	3	IV	NA	CR
18	F/63	non-GCB	0	2	IIB	NA	CR
19	M/81	GCB	2	3	III	Yes	PR
21	F/58	GCB	0	3	IV	Yes	PR
22	M/55	GCB	1	1	IV	No	CR
23	F/82	non-GCB	1	1	IIE	Yes	CR
24	M/36	non-GCB	0	NA	IV	NA	CR

Supplemental Table S2. Patient characteristics and LymphGen input and output features - core predictions

Sample ID	Outcome	Copy Number	BCL2 Translocation	BCL6 Translocation	Model	Subtype Prediction - core	Confidence BN2	Confidence EZB	Confidence MCD	Confidence N1	Confidence ST2	Confidence A53	BN2 Features Count	EZB Features Count	MCD Features Count	N1 Features Count	ST2 Features Count	A53 Features Count
UPN 1	CR - Alive	Available	No	Yes	Full	MCD/A53	0.70	0.04	**0.91**	0.08	0.00	**0.99**	3	1	6	0	0	6
UPN 2	PR - Deceased	Available	NI	NI	No Fusion	Other	0.27	0.04	0.00	0.08	0.00	0.00	2	1	1	0	0	0
UPN 3	CR - Alive	Available	No	No	Full	Other	0.04	0.04	0.00	0.08	0.04	0.86	1	1	2	0	2	4
UPN 4	CR - CNS relapse - Alive	Available	NI	NI	No Fusion	Other	0.18	0.76	0.00	0.08	0.63	0.00	2	3	0	0	5	0
UPN 7	CR - Alive	Available	NI	NI	No Fusion	A53	0.03	0.02	0.00	0.08	0.00	**0.94**	0	0	0	0	0	5
UPN 11	PR - Deceased	Available	NI	NI	No Fusion	BN2	**0.99**	0.04	0.00	0.08	0.01	0.00	5	1	1	0	1	1
UPN 12	CR - Alive	Available	No	Yes	Full	BN2	**0.99**	0.84	0.00	0.08	0.00	0.02	5	3	0	0	0	1

Confidence values that defined the core prediction are shown in bold.
NI = Not investigated

Supplemental Table 3. Patient characteristics and LymphGen input and output features - extended predictions

Sample ID	Outcome	Copy Number	BCL2 Translocation	BCL6 Translocation	Model	Subtype Prediction - Extended	Confidence BN2	Confidence EZB	Confidence MCD	Confidence N1	Confidence ST2	Confidence A53
UPN 1	CR - Alive	Available	No	Yes	Full	MCD/A53	0.70	0.04	**0.91**	0.08	0.00	**0.99**
UPN 2	PR - Deceased	Available	NI	NI	No Fusion	Other	0.27	0.04	0.00	0.08	0.00	0.00
UPN 3	CR - Alive	Available	No	No	Full	A53	0.04	0.04	0.00	0.08	0.04	**0.86**
UPN 4	CR - CNS relapse - Alive	Available	NI	NI	No Fusion	EZB	0.18	**0.76**	0.00	0.08	0.63	0.00
UPN 7	CR - Alive	Available	NI	NI	No Fusion	A53	0.03	0.02	0.00	0.08	0.00	**0.94**
UPN 11	PR - Deceased	Available	NI	NI	No Fusion	BN2	**0.99**	0.04	0.00	0.08	0.01	0.00
UPN 12	CR - Alive	Available	No	Yes	Full	BN2	**0.99**	0.84	0.00	0.08	0.00	0.02

Confidence values that defined the core and extended predictions are shown in bold.
NI = Not investigated
